# Comparison of physiological responses of Arabian striped hyaena (*Hyaena hyaena sultana*) to effective immobilisations with ketamine-medetomidine and ketamine-xylazine in (semi-) captive conditions

**DOI:** 10.7717/peerj.7326

**Published:** 2019-07-26

**Authors:** Abid Mehmood, Sadia Abid, Pavla Hejcmanová, Muhammad Arslan Asadi, Bilal Kabeer, Muhammad Jawad Jilani, Sadaf Bilal, Muhammad Waseem Ashraf

**Affiliations:** 1 Department of Animal Science and Food Processing, Faculty of Tropical AgriSciences, Czech University of Life Sciences, Prague, Czech Republic; 2 Department of Wildlife and Conservation Services, Sir Bani Yas Island, Barari Natural Resources, Abu Dhabi, United Arab Emirates

**Keywords:** Blood gas analysis, Immobilisation, Vital signs, Induction, Anaesthesia, Carnivores, Hyaena

## Abstract

Chemical immobilisation is an integral component for the conservation of wild animals and can be stressful if proper protocols are not administered. References on the immobilisation of Arabian striped hyaena (*Hyaena hyaena sultana*) are scarce. The current study was designed to evaluate the physiological and clinical responses of Arabian striped hyaena, immobilised with ketamine-medetomidine (KM) and ketamine-xylazine (KX); and to compare immobilisation effectiveness of the two combinations in a cross-sectional clinical study. A total of 15 (six males, nine females) (semi-) captive and adult Arabian striped hyaena with an average weight of 31.39 ± 0.36 kg were immobilised 50 times for annual vaccination and translocation purposes from January 2014 till March 2018 on Sir Bani Yas Island, United Arab Emirates. A total of 34 immobilisations were executed with (Mean ± SE) 2.27 ± 0.044 mg/kg ketamine and 0.04 ± 0.001 mg/kg medetomidine; while 16 with 4.95 ± 0.115 mg/kg ketamine and 0.99 ± 0.023 mg/kg xylazine. The drugs were remotely delivered intramuscular. The evaluation of physiological and clinical parameters included monitoring of vital signs through pulse oximetry, blood gas analysis of arterial blood through Istat blood gas analyser, and blood biochemistry and haematology. The quality of induction, anaesthesia and recovery was also assessed. Atipamezole (0.21 ± 0.003 mg/kg) was used to antagonise the effects of KM and 0.09 ± 0.003 mg/kg atipamezole or by 0.23 ± 0.006 mg/kg yohimbine for KX. Data were analysed using the general linear model and inferential statistics. KM was more effective in induction (scores; KM = 1.41 ± 0.10; KX = 1.31 ± 0.12), anaesthesia (KM = 1.00 ± 0.00; KX = 2.0 ± 0.0) and recovery (KM = 1.76 ± 0.15; KX = 2.69 ± 0.12) phases as compared to KX. There was a significant difference (*P* < 0.05) amongst the two combinations for anaesthesia time (KM = 59.5 ± 2.41; KX = 49.25 ± 1.31 min.), time to stand after reversal (KM = 4.91 ± 0.60; KX = 10.38 ± 1.48 min.) and full loss of the signs of anaesthetics (KM = 12.32 ± 1.37; KX = 21.25 ± 2.16 min.) along with rectal temperature (KM = 37.58 ± 0.29; KX = 36.00 ± 0.68 °C), pulse rate (KM = 50.46 ± 1.90; KX = 61.14 ± 2.79 beats/min), respiration rate (KM = 29.44 ± 0.99; KX = 23.80 ± 1.57 breaths/min.) and partial pressure of oxygen (KM = 89.59 ± 1.34; KX = 82.06 ± 3.92%). The blood oxygen saturation by oximeter indicated hypoxaemia in KX (82.06 ± 3.92), supported by the data from blood gas analyser. KM combination was more suitable for the immobilisation of Arabian striped hyaena, providing a better quality of induction, anaesthesia and recovery compared to KX. However, we strongly suggest further investigation to see the effects of oxygen supplementation for the compensation of hypoxaemia.

## Introduction

The Arabian striped hyaena (*Hyaena hyaena sultana*) is the most critical large scavenger found in the tropical grassland and woodland ecosystems (Kruuk, 1976). The striped hyena is a carnivore with a broad head, long ears and somewhat pointed muzzle. The body slopes down from head to tail bearing black stripes on pale or pale grey underfur (Rosevear, 1974). Hyaenas resemble dogs in their appearance, but the skull, teeth and other anatomical resemblances bring them closer to felines. Therefore, they are placed in separate family Hyaenidae in the suborder Feliformia (cats and cat-like carnivores) and are closely related to domestic cats than dogs (Prater, 1971; Wilson & Mittermeier, 2009; Hahn et al., 2014). *H. hyaena* is categorised as Near Threatened in the Red List of Threatened Species by IUCN (AbiSaid & Dloniak, 2015). *H. h. sultana* is distributed from Oman, Saudi Arabia, and the United Arab Emirates (UAE) until Yemen; in the UAE, it was last sighted in the wild in 1996 (Hellyer & Aspinall, 2005).

Chemical immobilisation and anaesthesia is an integral component of conservation, diagnostic and surgical procedures in wild animal species (Wack, 2003). Animal capture and handling are one of the most stress-inducing events for wild animals. The effects of stress on the blood haematology and biochemistry of the animal are directly related to the capture technique employed and are much reduced in chemical capture as compared to the physical capture (Marco & Lavin, 1999).

Ketamine hydrochloride has been widely used for the immobilisation of wild carnivores. It is used as an anaesthetic in combination with other drugs and has an eclectic range of benefits such as broader safety margin, low cost, high rate of absorption, lower cardiovascular and respiratory effects, and its international accessibility (Burroughs, 1993). However, ketamine has some disadvantages such as the requirement of a higher dose, excessive salivation, muscular hypertonicity and contractions with poor muscle relaxation, damage to retina and mydriasis, occasionally a transient and minor respiratory depression, rough inductions and recoveries, and non-availability of the antagonist (Adams, 2001). As the pharyngeal and laryngeal reflexes are not affected by ketamine, excessive salivation is not a threatening condition. However, if deemed necessary to avoid the possibility of aspiration pneumonia, salivary hypersecretion can be controlled by administration of atropine sulphate (Heinz et al., 2006; Thomas, 2013).

Ketamine combinations with α2-adrenergic agonists such as medetomidine or xylazine can reduce the dosage of ketamine, suppress its side effects and provide anxiolysis and muscle relaxation (Knight, 1980; Adams, 2001; Sinclair, 2003). Xylazine is an α2-adrenergic agonist that depresses the central nervous system, induces sedation and has a myorelaxation effect. The side effects of xylazine include regurgitation especially in carnivores, reduced blood pressure, heart rate and rectal temperature, excessive salivation, and may cause abortion if the female animal is pregnant (Knight, 1980; Shindle & Tewes, 2000; Rockhill et al., 2011). Medetomidine is a highly specific α2-adrenergic agonist that has ten times more affinity and 200 times more selectivity for adrenoceptors and induces elongated duration of analgesia and anaesthesia than xylazine (Sinclair, 2003). The undesired effects of medetomidine include hypertension frequently followed by peripheral hypotension, hypothermia, respiratory depression and bradycardia (Jalanka & Roeken, 1990). The effects of medetomidine and xylazine can be reversed using potent and selective α2-adrenoceptor antagonist such as atipamezole hydrochloride. The effects of xylazine can also be reversed using yohimbine hydrochloride (Jalanka & Roeken, 1990; Hahn et al., 2014).

Spotted hyaena have been immobilised successfully with ketamine at a dosage rate of 7–15 mg/kg (Smuts, 1973), xylazine one mg/kg with one mg/kg phencyclidine (Young & Whyte, 1973), zoletil four mg/kg (Van Jaarsveld & Skinner, 1991), xylazine 10.7 ± 1.9 mg/kg with ketamine 0.5 ± 0.1 mg/kg (Van Jaarsveld, McKenzie & Meltzer, 1984) and ketamine four to six mg/kg with xylazine one mg/kg (Hahn et al., 2014) in numerous studies. However, the references on the immobilisation of Arabian striped hyaena are scarce. In the present study, we investigated the two combinations of ketamine with xylazine and medetomidine and assessed the quality of induction, anaesthesia, and recovery stages along with the effects of these drugs on the physiological and clinical parameters through monitoring of vital signs, blood haematology, biochemistry, blood gas analysis and behavioural response of the animals to immobilisation.

## Materials and Methods

### Study area

The study was conducted in Sir Bani Yas Island, which is 250 km West of Abu Dhabi and is 87 km^2^ (8,700 hectares) in size. The entire island is protected; an Arabian Wildlife Park consisting of 4,100 ha which was developed in 2009. Roughly over 16,000 individuals belonging to various species inhabit the island, with 2.5 million planted trees providing a suitable habitat to the animals (Dhaheri et al., 2017).

### Study animals

A total of 15 (six males and nine females) (semi-) captive adult *H. h. sultana*, with an average weight and standard error of 31.39 ± 0.36 kilograms, were managed under conservation breeding project at the island. A total of 50 immobilisations (ketamine-medetomidine (KM): *n* = 34 and ketamine-xylazine (KX): *n* = 16) were performed on these 15 animals as part of routine animal management and veterinary interventions for vaccinations (necessary protocol for the whole collection) and translocations. Additionally, 18 immobilisations were performed for treatments from January 2014 till March 2018 on Sir Bani Yas Island, UAE (Table 1). The animals were free roaming in 4,100 ha park or were housed in enclosures of an average size of 0.57 ± 0.16 ha (mean ± SE). The animals and protocols (KM or KX) were randomly selected by using simple random sampling (Suresh, Suresh & Thomas, 2011). However, due to a shortage of xylazine during the study, more immobilisations had to be performed with KM combination. The 18 immobilisations of injured animals with KM combination, rated as ASAPS Class 2E according to American Society of Anaesthesiologists (Doyle & Garmon, 2018), were excluded from the analysis due to biased numbers of patients, as a single patient (out of four patients) received 12 out of 18 treatments and pre-anaesthesia excitement and trauma affecting the quality of induction, anaesthesia and recovery phases. All the immobilisations were performed according to the highest animal care and management standards and institutional animal health care and management plan (BFM-WL-CI/0017-2014). No patient was explicitly selected for study purposes; therefore, ethical committee approval was not required.

**Table 1:** Details of investigated animals and reasons for immobilisation of *Hyaena hyaena sultana* immobilised with Ketamine-Medetomidine and Ketamine-Xylazine combinations in (semi-) captive conditions.

Animal ID	Sex	Age	Average weight (kg) ± SE	Number of immobilisations	Health condition/reasons for immobilisation (*n*)
Aramis	Male	Adult	34.50 ± 3.37	3	HV
Arnold	Male	Adult	36.70 ± 3.20	5	TL (1); HV (4)
Athos	Male	Adult	30.85 ± 0.73	3	HV
Bravo	Male	Adult	31.10 ± 1.63	5	TL (1); HV (4)
Buwaytir	Male	Adult	29.85 ± 1.23	3	TL (1); HV (2)
Porthos	Male	Adult	30.17 ± 1.83	3	TL (1); HV (2)
Alpha	Female	Adult	28.83 ± 1.43	3	TL (2); HV (1)
Chips	Female	Adult	31.13 ± 1.01	4	TL (1); HV (3)
D’Artagnan	Female	Adult	30.70 ± 0.89	5	TL (1); HV (4)
Dopey	Female	Adult	30.83 ± 1.48	3	HV (3)
Gadget	Female	Adult	30.57 ± 1.43	3	HV
Luna	Female	Adult	32.43 ± 0.57	2	HV
Phiri	Female	Adult	29.65 ± 1.22	5	TL (2); HV (3)
Sirius	Female	Adult	34.00 ± 0.00	1	HV
Starsky	Female	Adult	31.25 ± 0.75	2	TL

**Note:**

TL, translocations; HV, health check and vaccination.

### Chemical restraint

The animals were darted either from a vehicle or a hiding trailer depending on the conditions, ensuring that the animal was not excited prior to the drug delivery. Due to the high temperatures in the region, all the immobilisations were either early morning or late evening. The ideal darting site was hindquarters. However, depending on the orientation of the animal, the neck area was also found suitable (Hahn et al., 2014). The intramuscular (IM) drug injection was executed through Dan-Inject CO_2_ Injection Rifle (No. 0471, Model JM) using a dart syringe of three ml with a plain (1.5 × 20 mm) Dan-Inject needle. To avoid data biasness, it was made sure that all injections were completely delivered IM so that a second dart or supplementary dosage was not required. It was made sure that the animals were not near their burrows while roaming in 4,100 ha area and the burrows were closed in the smaller enclosures to halt the entry of drugged animals into the burrows. In the two combinations, the drugs used were ketamine hydrochloride (Ketamil Injection), medetomidine hydrochloride (Ilium Medetomidine Injection), and xylazine hydrochloride (Ilium Xylazil—100) from Troy Laboratories, Australia. The target dosage in KM was 2.33 mg/kg and 0.04 mg/kg, while five mg/kg and one mg/kg for KX calculated from literature for hyaena species (Kock & Burroughs, 2012; Hahn et al., 2014). The drug dosages were calculated by visual weight estimation and previous weighing records of the animals according to Table 2, whereas the actual weight was measured using a portable weighing scale and actual administered drug dose was calculated (Fournier et al., 1995; Lescano et al., 2014). Once the procedure was finished, the effects of α2-adrenergic agonist were reversed using atipamezole hydrochloride (Ilium Atipamezole Injection; Troy Laboratories, Glendenning, NSW, Australia), or yohimbine hydrochloride (Reverzine^TM^ Injection; Bayer, Pymble, NSW, Australia). Xylazine is usually reversed with yohimbine, but it was not available during some of the immobilisations, and we had to work with the opportunity in line with institutional and veterinary requirements at those times. Therefore, partial reversals of KX combination were with atipamezole and partial with yohimbine. The observers recording vital signs, quality of anaesthesia, and taking and processing blood samples were double-blinded to avoid biases.

**Table 2:** Dosage of drug agonists and antagonists in two combinations for the immobilisation of *Hyaena hyaena sultana* immobilised with Ketamine-Medetomidine and Ketamine-Xylazine combinations in (semi-) captive conditions.

Combinations	Drugs	Concentration (mg/ml)	Mean dosage ± SE (mg/kg)	Standard dose (ml)
Ketamine + Medetomidine (KM)	Ketamine	100	2.27 ± 0.044	0.7
	Medetomidine	1	0.04 ± 0.001	1.3
	Atipamezole (Reversal)	5	0.21 ± 0.003	1.3
Ketamine + Xylazine (KX)	Ketamine	100	4.95 ± 0.115	1.5
	Xylazine	100	0.99 ± 0.023	0.3
	Atipamezole (Reversal)	5	0.09 ± 0.003	0.6
	(or) Yohimbine (Reversal)	10	0.23 ± 0.006	0.7

### Duration and quality of the anaesthesia

The time was recorded from the delivery of dart until induction and later anaesthesia and recovery. After the induction animal was approached silently and was checked for the state of anaesthesia by touching it through a bamboo stick. If there was no response, the animal was placed in a lateral position as they have a simple stomach, and in the lateral position, their respiration remains effective (Kock & Burroughs, 2012). The chemical restraint procedure was divided into three categories viz. induction, anaesthesia and recovery. For each category, times in minutes for different events were recorded. For induction, the times from injection of anaesthetics to the time of first sign of the drug taking effect (ataxia), sternal position, head down and completion of induction were recorded. Anaesthesia period was considered the duration in which animal had sufficient loss of body movements or sudden jerks, the absence of pedal and palpebral reflexes, no muscle tone and no response to an external stimulus such as injections and sample collection and was calculated as the time from the completion of induction till administration of the reversal. The reversal was administered when the animals exhibited the signs of drugs being metabolised and losing their effects, such as pedal of palpebral reflexes, ear twitching, and grunting sounds. For recovery, the times were recorded for the duration from the administration of reversal till first sign of consciousness (ear twitching, blinking of eyes, retraction of the tongue), lifting head up, going to sternal position and complete recovery (Fournier et al., 1995; Lescano et al., 2014). The quality of induction, anaesthesia and recovery were assigned a numerical score from 1 (excellent) to 4 (unsatisfactory) as described by Lescano et al. (2014). The description for each score is presented in Table 3.

**Table 3:** Scoring Table for Induction, Immobilisation Quality and Recovery for immobilisation of *Hyaena hyaena sultana* immobilised with KM and KX combinations (modified from Lescano et al., 2014).

Score	Quality	Induction	Anaesthesia	Recovery
1	Excellent	Quick and smooth induction; absence of uncoordinated movement, stereotypic reaction, ptyalism, vomiting, and discomfort at the injection site	The absence of body movement, pedal and palpebral reflexes, muscle tone, response to an external stimulus	Quick and smooth recovery; absence of uncoordinated movement, ptyalism, vomiting, quick retraction of the tongue
2	Good	Quick induction but resistance to loss of balance, slight ptyalism, pacing, and licking	Ear twitching, no muscle tone, occasional (<3 times) pedal and palpebral reflexes, occasional body twitching to an external stimulus	Quick recovery, slight struggle to balance, slight ptyalism, licking, and slightly weak coordination in hindquarters
3	Satisfactory	Moderate or Slow induction, pacing more than 50 m, ptyalism, slight discomfort at the injection site, panting and grunting	Ear and limb twitching, slight muscle tone, delayed pedal and palpebral reflexes, slight twitching to an external stimulus	Slow recovery, delayed retraction of the tongue, Ptyalism, struggle in standing and uncoordinated movements, erection of mane hair
4	Unsatisfactory	No induction or violent resistance, vomiting, panting, stereotypic pacing, grunting, excessive ptyalism, severe discomfort to the injection site and licking	Body movement, limb withdrawn, grunting sounds, increased muscle tone, blinking, twitching of ear, immediate pedal and palpebral reflexes, rapid response to an external stimulus	Delayed recovery, inability to retract tongue, gain balance and no palpebral reflexes, unable to stand, temporary loss of sensation in the hindquarter, shivering

**Note:**

The scoring for induction, immobilisation and recovery was assessed separately based on observations at each stage during an immobilisation procedure.

### Monitoring of the vital signs

During anaesthesia phase, the rectal temperature, pulse rate, respiration rate, non-invasive blood pressure, capillary refill time, and blood oxygen saturation (SpO_2_) were recorded at 5-min intervals (Janovsky et al., 2000; Lescano et al., 2014). Changes in body temperature were measured through rectal thermometer (NOVAMED Digital thermometer, Surrey, England, UK) and rectal thermometer probe of the pulse oximeter (Purescope Veterinary Patient Monitor IP-3000/4000 Series; Infunix Technology Co., Ltd., Seoul, South Korea). Pulse rate was monitored by cardiac auscultation and by a pulse oximeter to cross-verify by manual observations (Janovsky et al., 2000). Respiration rate was monitored manually through direct observation of the movement of the thoracic cavity. The blood oxygen level was measured through pulse oximeter with the probe placed at the tongue of the animals. Systolic and diastolic non-invasive blood pressure was measured through pulse oximeter by placing the cuff on the median palmar artery (proximal to the metacarpal pad) as instructed in the operational manual of the monitor (Infunix Technology Co., Ltd., Seoul, South Korea). Capillary refill time was assessed by pressing the oral mucosa through thumb and then counting the required seconds to regain its colour (Lescano et al., 2014).

### Blood gas analysis

The blood was taken from the femoral artery after 15 min into the anaesthesia and immediately analysed using I-Stat handheld blood gas analyser (iSTAT^®^ 1, Abbott Laboratories, Lake Bluff, IL, USA) and i-STAT^®^ CG8+ Cartridge. The variables that were measured included pH, partial pressure of carbon dioxide, partial pressure of oxygen (pO_2_), blood concentrations of sodium, potassium, glucose (Glu) and ionised calcium (iCa), total carbon dioxide, bicarbonate, base excess, haematocrit (Hct), haemoglobin (Hb), and haemoglobin oxygen saturation (sO_2_) (Lescano et al., 2014). Due to lack of available reference range for parameters mentioned above, feline range provided with the test kit was used as hyaenas are more closely related to felids, especially their physiological responses to xylazine and ketamine (Wilson & Mittermeier, 2009; Hahn et al., 2014; ABAXIS, 2018).

### Blood haematology and biochemistry analysis

Blood biochemistry and haematology was performed on the blood samples drawn from the jugular vein using 20 ml disposable syringes. Blood (six to eight ml) was collected in serum gel tubes for blood biochemistry, three to four ml in heparin lithium tubes for cortisol hormone, and three to four ml in EDTA K_3_ tubes for haematology. The blood in the serum gel and heparin tubes was centrifuged at 2,000 rpm for 3–5 min and 1,000 rpm for 2–3 min, respectively. The samples were either analysed using Abbott CELL-DYN^®^ 3700 Haematology Analyser or Abbott Architect c4000 clinical chemistry analyser; or through Central Veterinary Research Laboratory (paid services). The study variables included; red blood cells count (RBC), Hb, packed cell volume (PCV), mean cell volume, mean cell haemoglobin (MCH), platelets, white blood cell count (WBC), neutrophils, lymphocytes, monocytes, eosinophils, basophils, creatinine kinase, lactate dehydrogenase (LDH), calcium (Ca), phosphorus (PHOS), aspartate transferase (AST), alanine transferase (ALT), alkaline phosphate (ALP), total bilirubin, creatinine (CREA), blood urea nitrogen, total protein (TP), albumin, uric acid, triglycerides, cholesterol, α-Amylase, Glu, and cortisol. The parameters mentioned above were compared to the references ranges of striped hyaena provided in ZIMS by Species 360.

### Data analyses

The recorded data for vital signs (*n* = 34 for KM and *n* = 16 for KX); blood gas and clinical biochemistry analyses (*n* = 21 for KM and *n* = 16 for KX) were assessed through Shapiro–Wilk test for normal distribution and then were analysed through general linear model using STATISTICA software, with main effects, individuals as random, combination and sex were fixed.

## Results

All 50 immobilisation events were successful with both combinations at induction, anaesthesia and recovery phases. The quality of induction was between excellent and good with the mean score and standard error (MS ± SE) 1.41 ± 0.10 and 1.31 ± 0.12 in KM and KX, respectively. The quality of anaesthesia was excellent in KM (1.00 ± 0.00) as compared to KX, where it was scored as good (2.0 ± 0.0). The recovery of the animals in KM was between excellent and good (1.76 ± 0.15), whereas, the quality of recovery in KX was between good and satisfactory (2.69 ± 0.12), especially reversing with yohimbine (3.00 ± 00) as compared to atipamezole (2.50 ± 0.17).

The time variables of both combinations for different stages of immobilisation are provided in Table 4. There was a significant difference amongst the two combinations for anaesthesia time (*F* = 5.1; *P* < 0.05), time to stand after administration of reversal (*F* = 8.3; *P* < 0.05) and complete recovery (*F* = 7.9; *P* < 0.05). Moreover, there was a significant difference between the sexes in the time to lift the head (*F* = 4.2; *P* < 0.05) and time to stand after administration of reversal (*F* = 6.2; *P* < 0.05). Females took less time compared to males. However, we could not establish any reason why females were quicker to lift the head and stand compared to males, and there was no significant difference in time to full recovery between the sexes. The two reversing agents for KX showed a significant difference in time to full recovery (*F* = 529.0; *P* < 0.05).

**Table 4:** Time variables of various chemical restraint stages for *H. h. sultana* immobilised with Ketamine-Medetomidine and Ketamine-Xylazine combinations in (semi-) captive conditions.

Time variables	KM (n = 34)	KX (n = 16)	*F* statistics for factors with *P* < 0.05
	Mean ± SE (Minutes)	Mean ± SE (Minutes)	
Time to first sign (ataxia)^*^	4.56 ± 0.40	4.19 ± 0.33	–
Time of sternal position^*^	6.15 ± 0.65	5.75 ± 0.39	–
Time of head down^*^	6.80 ± 0.65	6.87 ± 0.43	–
Induction time^*^	10.12 ± 0.65	9.37 ± 0.45	–
Anaesthesia time	59.5 ± 2.41	49.25 ± 1.31	*F*_DRUG_ = 5.1
Time to first reversal sign^+^	1.94 ± 0.22	2.13 ± 0.35	–
Time to head up^+^	3.70 ± 0.56	4.13 ± 1.13	*F*_SEX_ = 4.2
Time to sternal position (Reversal)^+^	4.03 ± 0.54	5.63 ± 1.09	–
Time to stand^+^	4.91 ± 0.60	10.38 ± 1.48	*F*_DRUG_ = 8.3; *F*_SEX_ = 6.2
Time to complete recovery^+^	12.32 ± 1.37	21.25 ± 2.16	*F*_DRUG_ = 7.9

**Notes:**

* Time from administration of anaesthetics

+ Time from administration of reversal.

**Figure 1:**
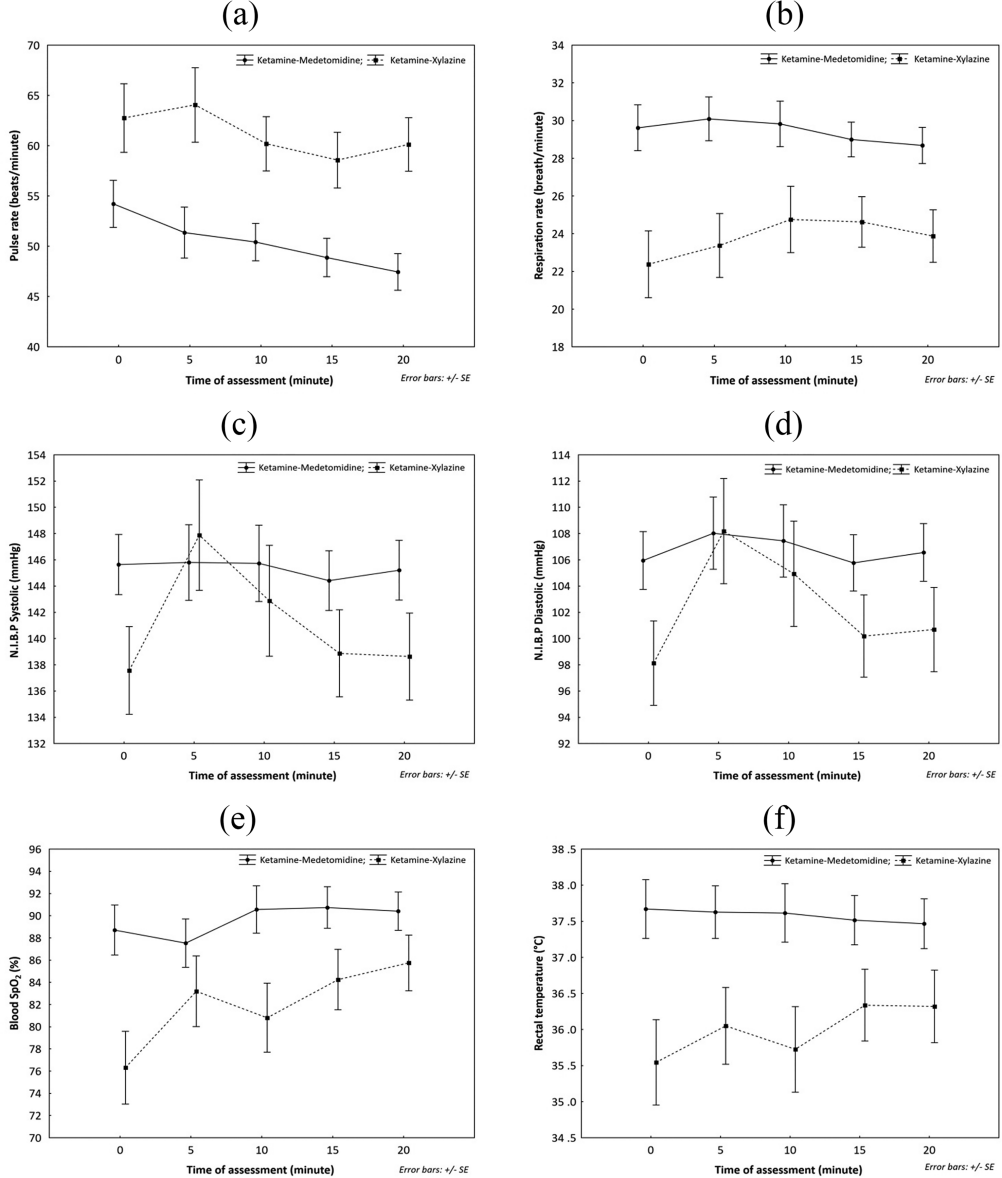
Comparison of mean variation of vital signs during anaesthesia of *Hyaena hyaena sultana* using combinations of Ketamine-Medetomidine and Ketamine Xylazine in (Semi-) Captive conditions; (A) pulse rate; (B) respiration rate; (C) N.I.B.P. systolic; (D) N.I.B.P. diastolic; (E) Blood SpO_2_; (F) rectal temperature.

The mean variations of pulse rate, respiration rate, systolic and diastolic non-invasive blood pressure, blood oxygen saturation and rectal temperature throughout anaesthesia are presented in Fig. 1. The capillary refill time for all immobilisations was less than 2 s. There was no significant difference in the vital signs between males and females (*P* > 0.05). However, Rectal temperature, pulse rate, respiration rate and blood oxygen saturation were significantly different (*F* = 7.5, 6.4, 6.8 and 4.6, respectively, with *P* < 0.05) between the combinations (Table 5).

**Table 5:** Comparison of physiological variables of vital signs and blood gas analysis during chemical restraint of *H. h. sultana* immobilised with KM and KX combinations in (semi-) captive conditions.

Vital sign variables	Units	Range	KM (*n* = 34) Mean ± SE	KX (*n* = 16) Mean ± SE	F statistics for factors with *P* < 0.05
Rectal temperature	°C	–	37.58 ± 0.29	36.00 ± 0.68	*F*_DRUG_ = 7.5
Pulse rate	Beats/min	–	50.46 ± 1.90	61.14 ± 2.79	*F*_DRUG_ = 6.4
Respiration rate	Breaths/min	–	29.44 ± 0.99	23.80 ± 1.57	*F*_DRUG_ = 6.8
SpO_2_	%	–	89.59 ± 1.34	82.06±3.92	*F*_DRUG_ = 4.6
N.I.B.P (Systolic)	mmHg	–	145.36± 2.13	141.16±3.66	–
N.I.B.P (Diastolic)	mmHg	–	106.74± 1.97	102.43± 3.50	–
		Range*	**KM (*n* = 21)**	**KX (*n* = 16)**	
pH	–	7.25–7.40	7.28 ± 0.01	7.26 ± 0.01	–
pCO_2_	mmHg	33.0–51.0	45.94 ± 1.02	46.79 ± 2.08	–
HCO_3_^−^	mmol/l	13.0–25.0	21.73 ± 0.67	22.22 ± 0.67	–
TCO_2_	mmol/l	16–25	22.81 ± 0.65	23.19 ± 0.61	–
BE	mmol/l	(−5)–(+2)	−5.52 ± 0.69	−5.94 ± 0.38	–
pO_2_	mmHg	90–110	53.00 ± 2.54	44.44 ± 3.08	*F*_DRUG_ = 5.9
sO_2_	%	>90	80.57 ± 2.39	69.44 ± 5.09	–
Na^+^	mmol/l	147–162	144.38 ± 0.25	143.50 ± 0.43	–
K^+^	mmol/l	2.9–4.2	4.35 ± 0.07	4.17 ± 0.08	–
iCa^++^	mmol/l	1.20–1.32	1.43 ± 0.01	1.45 ± 0.02	–
Hct	% PCV	24–40	38.33 ± 0.96	39.88 ± 0.93	–
Hb	g/dl	8.0–13.0	12.98 ± 0.32	13.21 ± 0.31	–

**Notes:**

SE for measurements of rectal temperature, pulse rate and respiration rate in an animal immobilised once (*n* = 1) indicates SE of five repeated/consecutive measurements during one immobilisation event. pH, potential of hydrogen; pCO_2_, partial pressure of carbon dioxide; pO_2_, partial pressure of oxygen; BE, base excess; HCO_3_, bicarbonate; TCO_2_, total carbon dioxide; sO_2_, oxygen saturation; Na^+^, sodium; K^+^, potassium; iCa^++^, ionised calcium; Hct, haematocrit; Hb, haemoglobin.

* Feline range from I-Stat reference manual for CG8+ Cartridge.

The blood gas analysis results showed no statistically significant difference between sexes or drug combinations except pO_2_ that had statistically significant difference among combinations (*F* = 5.9, *P* < 0.05). The comparison of variables from blood gas analysis to the range value of felines suggested that most of the variables remained within described range except ionised Calcium (iCa^++^) which was high; pO_2_ and sO_2_ were low.

The results of the clinical variables are given in Table 6. The statistical analysis of the variables between sexes showed a significant difference in platelet count, WBC, LDH and cortisol levels (*P* < 0.05). There was also a statistically significant difference between the combinations for MCH, PHOS, ALT, ALP, CREA, TP, Glu, α-Amylase, and cholesterol (*P* < 0.05). The mean values were compared with the range of values of striped hyaena extracted from ZIMS by Species 360, to assess the condition of the animals. All the variables were within the described range (Table 6).

**Table 6:** Comparison of clinical variables during chemical restraint of *H. h. sultana* immobilised with Ketamine-Medetomidine and Ketamine-Xylazine combinations in (semi-) captive conditions.

Variables	Units	Range*	KM (*n* = 21)	KX (*n* = 16)	*F* statistics for factors with *P* < 0.05
			Mean ± SE	Mean ± SE	
RBC	10^12^ cells/l	4.54–9.80	7.38 ± 0.13	7.11 ± 0.21	–
Hb	g/dl	9.8–17.6	15.39 ± 0.27	14.72 ± 0.43	–
PCV	l/l	–	0.45 ± 0.01	0.43 ± 0.02	–
MCV	fl	8.3–73.4	61.20 ± 0.77	60.21 ± 0.91	–
MCH	pg	15.8–24.6	21.29 ± 0.16	20.56 ± 0.20	*F*_DRUG_ = 6.04
MCHC	g/dl	28.4–38.1	34.70 ± 0.60	34.21 ± 0.68	–
PLT	10^9^/l	–	223.33 ± 6.3	210.75 ± 9.26	*F*_SEX_ = 6.05
IRON	μmol/l	–	17.9 ± 1.04	18.94 ± 1.37	–
WBC	10^9^/l	5.4–17.1	8.15 ± 0.22	7.15 ± 0.40	*F*_SEX_ = 13.7; *F*_INDIV_ = 2.6
NEU	%	53.6–88.5	70.30 ± 1.70	64.94 ± 1.83	–
LYM	%	6.9–38.0	25.57 ± 1.67	30.42 ± 2.10	–
MONO	%	0.9–10.6	3.59 ± 0.31	2.79 ± 0.25	–
EOS	%	0.0–8.0	2.21 ± 0.62	1.81 ± 0.54	–
BASO	%	0.0–3.6	0.02 ± 0.01	0.11 ± 0.07	–
CK	U/l	53–567	143.8 ± 10.9	262.0 ± 73.38	–
LDH	U/l	394–1810	579.8 ± 19.4	585.25 ± 28.18	*F*_SEX_ = 7.4
Ca	mmol/l	2.0–2.8	2.48 ± 0.02	2.44 ± 0.02	*F*_INDIV_ = 3.08
PHOS	mmol/l	0.60–1.70	1.48 ± 0.04	1.26 ± 0.03	*F*_DRUG_ = 28.8
AST/GOT	U/l	41–113	102.3 ± 7.16	99.56 ± 7.48	–
ALT/GPT	U/l	21–91	46.57 ± 2.54	37.25 ± 3.87	*F*_DRUG_ = 8.08
ALP	U/l	10.0–48.0	15.05 ± 1.09	12.88 ± 0.49	*F*_DRUG_ = 4.9
TBIIL	μmol/l	0.0–6.8	1.11 ± 0.10	1.38 ± 0.09	–
CREA	μmol/l	62–150	82.76 ± 2.94	92.56 ± 4.52	*F*_DRUG_ = 5.4
BUN	mmol/l	3.9–13.4	11.89 ± 1.01	11.54 ± 0.47	–
TP	g/l	53–74	67.52 ± 0.64	64.94 ± 0.44	*F*_DRUG_ = 9.6
ALB	g/l	15–32	29.95 ± 0.49	30.63 ± 0.43	–
Glu	mmol/l	3.5–12.1	8.03 ± 0.46	9.99 ± 0.60	*F*_DRUG_ = 4.4
α-Amylase	U/l	346–911	694.5 ± 22.3	519.13 ± 21.80	*F*_DRUG_ = 15.9
Cholesterol	mmol/l	3.8–11.0	5.16 ± 0.22	4.16 ± 0.15	*F*_DRUG_ = 13.7; *F*_INDIV_ = 2.6
UA	μmol/l	0–36	9.48 ± 0.44	8.06 ± 0.49	–
TG	mmol/l	0.4–2.2	1.44 ± 0.23	1.22 ± 0.07	–
Cortisol	nmol/l	–	477.4 ± 43.9	475.13 ± 54.23	*F*_SEX_ = 8.9

**Note:**

* Reference range for striped hyaena extracted from ZIMS by Species 360. RBC, red blood cell count; Hb, haemoglobin; PCV, packed cell volume; MCV, mean cell volume; MCH, mean cell haemoglobin; PLT, platelets; IRON, iron; WBC, white blood cell count; NEU, neutrophils; LYM, lymphocytes; MONO, monocytes; EOS, eosinophils; BASO, basophils; CK, creatinine kinase; LDH, lactate dehydrogenase; Ca, calcium, PHOS, phosphorus; AST, aspartate transferase; ALT, alanine transferase; ALP, alkaline phosphate; TBIL, total bilirubin; CREA, creatinine; BUN, blood urea nitrogen; TP, total protein; ALB, albumin; Glu, glucose; UA, uric acid; TG, triglycerides.

## Discussion

Chemical restraint with KX and KM combinations and their effects on the physiological and clinical parameters of Arabian striped hyaena are reported for the first time in the current study. With both combinations, the immobilisation was achieved rapidly for handling and execution of desired procedures such as vaccination, translocation or treatment according to described dosages (Table 2). It is incredibly essential that carnivores especially hyaenas assume quick induction due to their affinity to enter a burrow, hide, run deep into the forest or mountainous area, or intraspecific aggression and safety of both humans and animals. Quick induction for the immobilisation of wild animals facilitates monitoring of animals, reduction of injuries, stress, hyperthermia; and chances of animal escape or hiding (Hahn et al., 2014; Barros et al., 2018).

Ketamine is regarded as a general anaesthetic that has analgesic activity and no cardiopulmonary depression but has poor muscle relaxation and high excitatory state in animals with excessive salivation when administered alone. The amnestic and anaesthetic effects of ketamine are caused due to overstimulation and disruption of the nervous system. Ketamine alone is not recommended, especially in canids due to its over-excitatory effects and prolonged recoveries (Ward et al., 2006).

We required a lower dosage of ketamine than recommended (2.5–3.0 mg/kg) for carnivores with KM (Jalanka & Roeken, 1990). The actual dosage for ketamine in the current study for KM was 55% lower than the KX, suggesting that medetomidine is highly effective in enhancing the potency of ketamine. It could be attributed to the high affinity and selectivity of medetomidine to adrenoceptors than xylazine (Sinclair, 2003). Therefore, a lower dosage of ketamine was required that enhanced the quality of immobilisation by lowering the side effects of ketamine as well as enhancing reversal quality as ketamine does not have a reversal. Studies on snow leopard also suggested a reduction of ketamine doses up to 25% (Jalanka & Roeken, 1990).

The addition of ketamine also enhances the effect of medetomidine, which produces sedation depending on its dose and requires high dosage for complete immobilisation. The dosage of medetomidine (0.04 ± 0.001 mg/kg) was lower than the recommended dosage (0.06–0.1 mg/kg) for carnivores (Jalanka & Roeken, 1990) but was within the recommended dose for hyaena (Kock & Burroughs, 2012). Moreover, the dosage of ketamine in KX was closer (4.95 ± 0.115 mg/kg and) in the current study compared to that used in hyaenas (5–10 mg/kg) (Kock & Burroughs, 2012). The xylazine dose (0.99 ± 0.023 mg/kg) in our combination was within the dosage range of 0.5–1.0 mg/kg for spotted hyaena and 1.0 mg/kg for jaguar (Burroughs, 1993; Kock & Burroughs, 2012; Bharathidasan et al., 2014).

Due to high potency, suppression of norepinephrine in the nervous system, reduced emetic effects, longer sedative and analgesic duration of medetomidine, it is preferred over xylazine (Paddleford & Harvey, 1999). The pharmacokinetics of medetomidine reveal its rapid absorption and distribution due to its high lipophilic characteristics (Jalanka & Roeken, 1990). KM combination was found more effective in anaesthesia and recovery phases compared to KX combination according to the scoring system provided in Table 3.

The induction with both combinations was between excellent and good, giving smooth induction, the absence of stereotypic reactions, with no to little ptyalism and resistance to loss of balance. However, there was a single case of vomiting with KX that can be related to xylazine being emetic in Hyaenidae and other carnivores (Bufalari et al., 2007; Hahn et al., 2014). The studies on donkeys, captive cougars, Southern river otters, free-ranging African lions, and marmosets had excellent to good scores using ketamine with medetomidine or other drugs such as xylazine (Selmi et al., 2004a; Soto-Azat, Reyes & Medina-Vogel, 2004; Wenger et al., 2010; Bakker et al., 2013; Lescano et al., 2014; Maney et al., 2018). The quality and depth of the anaesthesia were excellent with KM combination providing deep anaesthesia, no body movements or sudden jerks, the absence of pedal and palpebral reflexes, no muscle tone and no response to an external stimulus such as injections and sample collection. It could be attributed to the clinical analgesic, sedative and myrelaxation properties of medetomidine in felids and canids (Jalanka & Roeken, 1990). The good quality of anaesthesia was similarly reported in marmosets using KM, wild dogs using KM with atropine, and wild leopards using KX (Ward et al., 2009; Belsare & Athreya, 2010; Bakker et al., 2013). On the other hand, KX combination provided good anaesthesia with occasional ear and body twitching, pedal and palpebral reflexes to the external stimuli (Mulder, 1978; Ward et al., 2006). Occasional body movements are associated with the effects of ketamine when used in combination with xylazine. With both combinations, good myorelaxation was achieved to even perform dental examination (Mulder, 1978; Jalanka & Roeken, 1990; Ward et al., 2006). The average anaesthesia time in both combinations was higher as compared to the described useful anaesthesia time (Jalanka & Roeken, 1990). The study on Golden-headed tamarinds, however, showed that the combination of dexmedetomidine with ketamine provided higher quality and duration of anaesthesia compared to KM (Selmi et al., 2004b).

Residual motor impairment is apparent when a higher dosage of ketamine in KX and KM combinations are used in carnivores, leading to ataxic recoveries (Jalanka & Roeken, 1990). Recovery in KM combinations was between excellent and good. The animals were quick to recover, without vomiting, and with quick retraction of the tongue, slight struggle to balance, slight ptyalism and occasionally weak coordination in hindquarters. Due to the lower dosage of ketamine in KM, the animals were able to recover smoothly and quickly. The recovery in KX combination was mostly satisfactory providing slow recovery, delayed retraction of the tongue, ptyalism, weak coordination or uncoordinated movements and erection of mane.

The reversals with atipamezole were more effective as compared to yohimbine, which coincides with the study of Jalanka & Roeken (1990), who described a rapid reversal with atipamezole; as it has a high alpha-2/alpha-1selectivity ratio of 8,526 compared to 40 of yohimbine. Moreover, yohimbine is known only to partially antagonise the effects of xylazine, leading to delayed recoveries (Paddleford & Harvey, 1999). Atipamezole also showed a significant difference in time to full recovery and produced shorter recovery times compared to yohimbine. The times to arousal or first reversal sign with both combinations were markedly shorter than that in snow leopards (9 min) (Jalanka & Roeken, 1990). It is essential in wild conditions where the post-anaesthetic period for animals is very crucial as they can be a victim of aggression from other conspecifics that reversal is quick and smooth (Rockhill et al., 2011).

The respiration rate in KX was lower than KM; as ketamine causes dose-dependent respiratory depression due to an imbalance of muscarinic—nicotinic cholinergic activity of the brain centre (Jalanka & Roeken, 1990). The respiration was occasionally shallow and fast during the middle of anaesthesia compared to the whole duration in KX, and with time, the respiratory rate decreased in both combinations. The respiratory depression is associated with α2-adrenergic agonist and ketamine may ameliorate the respiratory depression when used with medetomidine (McKenzie et al., 1993; Fernandez-Moran et al., 2001). KM provided a stable respiratory pattern throughout the immobilisation.

Both xylazine and medetomidine are known to cause initial oxygen depression and increased CO_2_ and subsequent compensation in cats and snow leopards (Jalanka & Roeken, 1990). The blood oxygen saturation monitored by oximeter indicated a significant difference between the combinations with KX showing signs of hypoxaemia. The blood gas analysis also indicated severe hypoxaemia with KX combination, although, KM was also below the described range. Oxygen supplementation is essential and useful intervention when working with KX and KM on Arabian hyaenas. Blood gas analysis revealed mild metabolic acidosis. In wild felids, the metabolic acidosis is supposed to be normal due to their high dietary protein intake (Fahlman et al., 2005; Wenger et al., 2010).

Hypoxaemia is a complication commonly associated with wildlife immobilisation, especially when animals are breathing normal air (West, Heard & Cauklett, 2007). α2-adrenergic agonists are known to depress the respiratory centres and sensitivity to carbon dioxide in many species. The administration of xylazine has shown central hypoxaemia due to pulmonary alterations (Celly et al., 1997). Hypoxaemia can be a serious complication if coupled with hyperthermia and may lead to the death of the animal if not compensated (West, Heard & Cauklett, 2007). However, there was no case of hyperthermia recorded with studied combinations.

The pattern of rectal temperature fluctuation during the anaesthesia (Fig. 1) reveals that the temperature starts to decrease in animals sedated with KM and slightly increases in KX which coincides with the reported disadvantages of xylazine that causes loss of thermoregulatory control and causes hyperthermia (Jalanka & Roeken, 1990; Fernandez-Moran et al., 2001). In the current study, no case of hyperthermia was recorded, and the rectal temperature in both combinations was within the reported range (Hahn et al., 2014; Sahu, Sahoo & Mohapatra, 2018). It is incredibly essential to administer KX when the external temperatures are not high, and animals are not subjected to undue physical exertion and stress; as external temperatures and prolonged chasing before dart can cause hyperthermia (Jalanka & Roeken, 1990).

Bradycardia is an intrinsic symptom with the agonist of α2-adrenoceptors, especially medetomidine (Jalanka & Roeken, 1990; Fernandez-Moran et al., 2001; Wenger et al., 2010). The heart rate was significantly lower in KM as compared to KX, but the means with both combinations were within the described range for hyaenas. Both combinations showed slight signs of bradycardia during the later phases of immobilisation, where the heart rate decreased gradually but not below the normal range. When ketamine is used with xylazine, it suppresses the bradycardic effect of xylazine through cardiotonic action reconciled by vagolytic action (Bharathidasan et al., 2014). The administration of anticholinergic drugs such as atropine is reported to reduce bradycardia and salivation in hyaenas when immobilised with KX (Hahn et al., 2014). However, in the current study, we did not administer atropine. Additionally, ventricular arrhythmias are reported in dogs and wolves when atropine is used with KM (Kreeger, 1996). Another prominent effect of medetomidine is hypotension that is marked with an initial increase and then hypo- or normotension that was also observed in the current study (Jalanka & Roeken, 1990).

Although, medetomidine is known for its adrenolytic properties where PCV decreases due to pooling of RBCs in the spleen (Jalanka & Roeken, 1990); the erythrocytes, PCV and Hct values were within the prescribed range for striped hyaena in the current study. α2-adrenergic insulin inhibition in the beta cells of the pancreas and higher production of Glu in the liver frequently causes hyperglycaemia with medetomidine. Moreover, in tigers higher dosage of xylazine caused hyperglycaemia (Jalanka & Roeken, 1990). No hyperglycaemia was observed in our studies. The clinical analysis of blood also showed that there was no chronic kidney or liver disorder as ketamine is not recommended for renal or hepatic dysfunctional patients (Jalanka & Roeken, 1990). Cortisol levels were significantly higher in females. In spotted hyaena, the cortisol levels in adult or dominant females are usually higher than males and female cubs (Van Jaarsveld & Skinner, 1992). The mean cortisol level for both sexes was higher compared to the reported values for spotted hyaena (87.96 ± 74.03 ng/ml and 101.83 ± 71.79 ng/ml for males and females, respectively). It suggests a response to immobilisation stress during the initial phases of the immobilisation. Due to unavailability of samples at the later stages of immobilisation, it is difficult to assess whether the initial stress to capture was suppressed or not (Van Jaarsveld & Skinner, 1992; Sheriff et al., 2011).

The limitations of the study included lack of data on effects of oxygen supplementation to the tested combination for compensation of hypoxaemia, and samples for cortisol at later stages of immobilisation, and unavailability of yohimbine and xylazine during the study that led to more immobilisations with KM.

## Conclusions

In conclusion, we found KM combination more suitable for the immobilisation of Arabian striped hyaena, providing better quality and timings of induction, anaesthesia and recovery as compared to KX. KX also provided, in general, adequate immobilisation of the animals and can be alternatively used with the provision of supplementary oxygen, as complication of hypoxaemia was common in both combinations. However, certain other parameters with KX, especially time to full recovery can be critical if there are other animals around and can harm the recovering individual. We found severe hypoxaemia with KX, although there was hypoxaemia in KM to some extent. It urges a further study with the provision of supplementary oxygen to assess its effectiveness in the reduction of hypoxaemia. Other limitations included unavailability of yohimbine and xylazine during the study that led to more immobilisations with KM.

## Additional Information and Declarations

### Data Availability

The following information was supplied regarding data availability: Mehmood, Abid (2019): assessment data. figshare. Dataset. https://doi.org/10.6084/m9.figshare.8174180.v2 Mehmood, Abid (2019): Vital signs 19092018.xlsx. figshare. Dataset. https://doi.org/10.6084/m9.figshare.7729535.v2.
